# Urinary tract infections: a retrospective cohort study of (mis)matching antimicrobial therapy and clinical outcome among Finnish adults

**DOI:** 10.1093/jacamr/dlae188

**Published:** 2024-11-26

**Authors:** Anu Patjas, T Sakari Jokiranta, Anu Kantele

**Affiliations:** Department of Infectious Diseases, Meilahti Vaccine Research Centre, MeVac, University of Helsinki and Helsinki University Hospital, P.O. Box 700, FI-00029 HUS Helsinki, Finland; Human Microbiome Research Unit, University of Helsinki, Haartmaninkatu 3, FI-00290 Helsinki, Finland; Centre of Excellence in Antimicrobial Resistance Research, University of Helsinki, Haartmaninkatu 3, FI-00290 Helsinki, Finland; Medicum, University of Helsinki, Haartmaninkatu 3, FI-00290 Helsinki, Finland; Department of Microbiology and Genetics, Vita Laboratories Ltd, Laivakatu 5 F, FI-00150 Helsinki, Finland; Department of Infectious Diseases, Meilahti Vaccine Research Centre, MeVac, University of Helsinki and Helsinki University Hospital, P.O. Box 700, FI-00029 HUS Helsinki, Finland; Human Microbiome Research Unit, University of Helsinki, Haartmaninkatu 3, FI-00290 Helsinki, Finland; Centre of Excellence in Antimicrobial Resistance Research, University of Helsinki, Haartmaninkatu 3, FI-00290 Helsinki, Finland

## Abstract

**Objectives:**

With the global spread of antimicrobial resistance, treating urinary tract infections (UTIs) is becoming more challenging. Clinical data on UTI outcomes are scarce in cases with antimicrobial treatment mismatching the uropathogens’ *in vitro* susceptibility profiles. We explored the association of (mis)matching antimicrobial treatment and clinical outcomes among patients with either ESBL-producing Enterobacterales (ESBL-PE) or non-ESBL-PE identified in urine samples.

**Patients and methods:**

In 2015–2019, we recruited 18–65-year-old patients with laboratory-confirmed, community-acquired ESBL-PE (*n* = 130) or non-ESBL-PE (*n* = 187) UTI. Our study involved collecting data on *in vitro* susceptibility profiles, antimicrobial therapy (microbiological match/mismatch) and clinical outcomes, and a follow-up of relapses/reinfections.

**Results:**

Non-beta-lactam co-resistance was found more frequent among ESBL-PE than non-ESBL-PE isolates. The initial antimicrobial matched the *in vitro* susceptibility for 91.6% (164/179) of those with non-ESBL-PE and 46.9% (38/81) with ESBL-PE UTI (*P* < 0.001). The clinical cure rates in the non-ESBL-PE and ESBL-PE UTI groups were 82.6% (142/172) and 62.2% (74/119) (*P* < 0.001) for all, 87.3% (131/150) and 83.3% (30/36) for those treated with matching antimicrobials, and 33.3% (5/15) and 41.9% (18/43) for those given mismatching antimicrobials, respectively. Mismatching antimicrobial therapy was not associated with relapse/reinfection over the 3-month follow-up (*P* = 0.943).

**Conclusions:**

In our data, (mis)matching microbiological susceptibility is only partially associated with the clinical outcome of UTI: microbiological matching appears to predict clinical cure better than mismatching predicts clinical failure.

## Introduction

Urinary tract infections (UTIs) rank the most common bacterial infections encountered in primary health care,^1^ with half of the female population experiencing at least one episode by 32 years of age.^2^ Over the past decades, the proportions of ESBL-producing Enterobacterales (ESBL-PE) have gradually grown in clinical urine isolates,^3^ posing more challenges in treating UTI.

International guidelines advise antimicrobials, such as trimethoprim/co-trimoxazole, nitrofurantoin, pivmecillinam, or fosfomycin, for first-line therapy against uncomplicated cystitis.^4,5^ For pyelonephritis and other upper UTIs, fluoroquinolones, co-trimoxazole or broad-spectrum beta-lactams are recommended.^4,5^ However, resistance against these antimicrobials is increasing among ESBL-PE and other urinary pathogens^3,6^ even in countries with low antimicrobial resistance (AMR) prevalence.^6,7^

The growing AMR prevalence indicates a growing risk of mismatching between *in vitro* susceptibility and empiric antimicrobial therapy. Research into the impact of such mismatching on the UTI outcome remains scarce. There is one investigation reporting no association between discordant antibiotic treatment and clinical outcome^8^ and three others describing an imperfect association.^9–11^ None look separately at ESBL-PE UTI patients.

We utilized the low AMR prevalence setting in a Nordic country with the objective to explore the impact of mismatching by investigating (i) resistance among urinary ESBL-PE and non-ESBL-PE; (ii) matching/mismatching between uropathogens’ *in vitro* susceptibility and initially prescribed antimicrobials (microbiological matching); (iii) clinical outcome (cure/failure) associated with individual antimicrobials; and (iv) compatibility between microbiological matching and clinical outcome.

## Patients and methods

### Participants and study design

**Figure 1.**
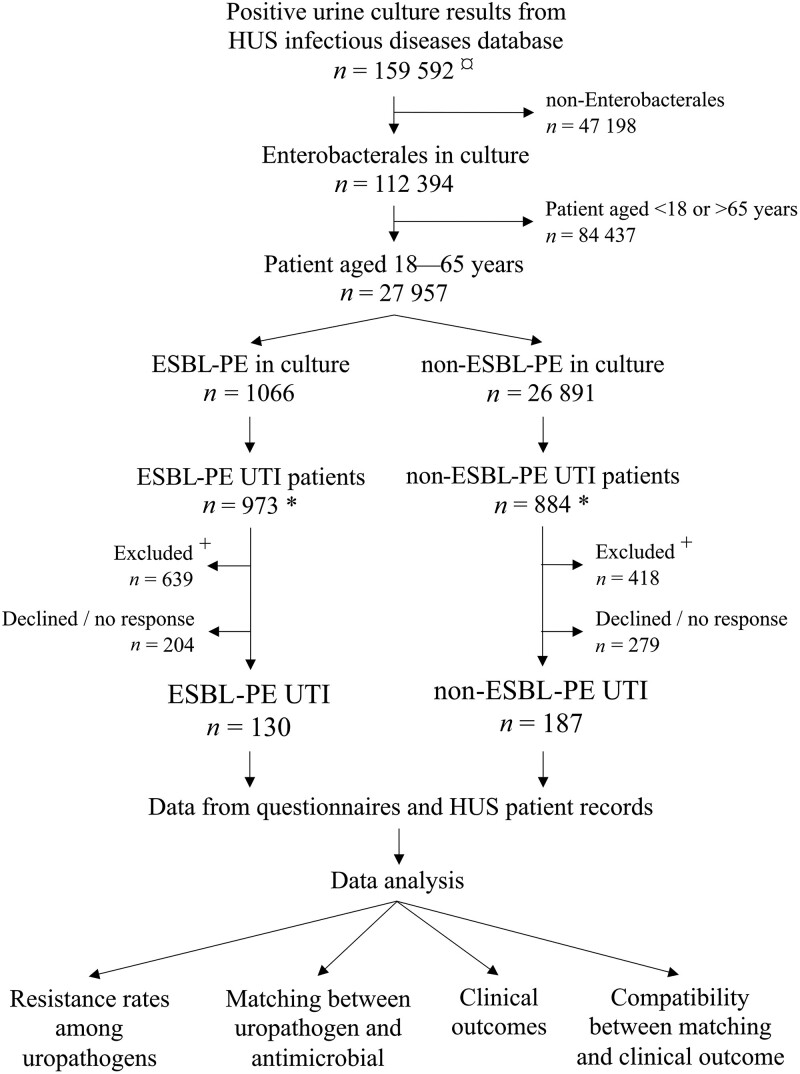
Selection of urinary samples with ESBL-PE and non-ESBL-PE findings, recruitment of 18–65-year-old participants in the two groups, and the schema of the various analyses conducted. ¤Over nine data retrievals, each covering urine samples from the past three months. *Deceased patients and multiple samples per patient removed; compared to ESBL-PE UTI patients, we selected a respective number of non-ESBL-PE UTI patients. ^+^The following exclusion criteria were applied: (i) previous ESBL-PE colonization (*n* = 263), (ii) residency in a nursing home (*n* = 42), (iii) hospitalization for more than 24 h during past month (*n* = 36), (iv) permanent residency abroad during past year (*n* = 8), (v) inability to participate (e.g. language barrier, aphasia or dementia) (*n* = 162), (vi) lack of contact information in HUS database or not reached (*n* = 519), and (vii) asymptomatic bacteriuria unless patient was pregnant (*n* = 27).

Data for the present study were initially collected as part of a three-arm case-control study investigating risk factors for ESBL-PE UTI.^12^ The Helsinki University (HUS) Ethical Committee approved the study protocol (408/13/03/00/2015); all patients provided written informed consent. Between 1 December 2015 and 31 May 2019, we retrieved information on patients with clinical urine samples collected from SAI, the infectious diseases database of HUS (Figure 1). We included all 18–65-year-old individuals with recent (≤3 months) urinary ESBL-PE, and a corresponding number of individuals with non-ESBL-PE findings. All potential participants were contacted by phone. Exclusion criteria covered asymptomatic bacteriuria (unless pregnant), prior ESBL-PE colonization/infection and suspected healthcare-acquired UTI.

Eligible patients were requested to fill in questionnaires at recruitment and after a 3-month follow-up. The questionnaires were completed on paper, electronically or by phone interview, depending on participants’ preferences. The initial questionnaire dealt with medical history, symptoms and treatments, covering past and index UTI episodes, while the follow-up questionnaire focused on recurrences occurring within 3 months of index episode. Additional data on antimicrobial regimens and blood cultures were retrieved from patient records by a separate consent.

### Exposure: microbiological matching/mismatching

The main variable of interest was matching (uropathogen sensitive *in vitro* to initial antimicrobial used) versus mismatching (uropathogen resistant *in vitro* to initial antimicrobial used) antimicrobial treatment. Individuals lacking data on the initial antimicrobial regimen were excluded from the matching analyses, unless the uropathogen was susceptible to all the primary panel antimicrobials (cephalexin, cefuroxime, trimethoprim, mecillinam, nitrofurantoin, ciprofloxacin), categorizing the treatment as matching.

For those having ESBL-PE with intermediate susceptibility (I) to pivmecillinam, the standard treatment of 200 mg three times a day^13^ was categorized as mismatching, and 400 mg three times a day as matching, according to recommendations.^14^ ESBL-PE UTI patients with no data on pivmecillinam dosage were excluded from the matching analyses.

For patients with co-infections, the matching analyses followed the main pathogen’s susceptibility (listed first in the standard laboratory report). For the compatibility analyses between microbiological matching and clinical outcome, differing matching results between uropathogens led to exclusion.

### Outcome: clinical failure

The primary outcome was the proportion of episodes resulting in clinical failure. As clinical failure we considered persisting symptoms at the time of the first questionnaire or receipt of a second antimicrobial regimen for the index UTI episode; routine transition from intravenous to peroral therapy was not considered as failure. Asymptomatic pregnant participants, individuals not receiving antimicrobial therapy and those whose symptoms resolved spontaneously were excluded from the clinical failure analyses.

The secondary outcome was the proportion of episodes with relapse/reinfection. As relapse/reinfection we considered each new UTI episode occurring after clinical cure over the 3-month follow-up. No urinary samples were required for diagnosis of relapse/reinfection (see UTI definition below).

### Urinary tract infections and recurrence

UTI was defined by symptoms of cystitis or upper UTI (pyelonephritis/urosepsis), as per UTI guidelines,^4,5,13^ and a concomitant bacterial growth ≥10^5^ cfu/mL. Afebrile (<38.0°C) cases were categorized as cystitis, and febrile (≥38.0°C) as pyelonephritis or urosepsis (same pathogen in urine and blood cultures).^1^

Recurrent UTI was defined according to international guidelines^5,15^: two or more episodes in the past 6 months or three or more in the past 1 year, including the index episode.

### Empiric antimicrobials recommended for UTI in Finnish guidelines

The Finnish UTI guidelines^13^ advise as the first-line empirical treatment for cystitis trimethoprim, co-trimoxazole (men only), nitrofurantoin, pivmecillinam (women only) or fosfomycin, and for upper UTI fluoroquinolones or cefuroxime.

### Identification and antimicrobial susceptibility of uropathogens

The uropathogens were identified and antimicrobial susceptibility was tested as part of routine clinical microbiology practices at the HUS Diagnostic Center. Urine samples were cultured on CHROMagar Orientation agar plates (CHROMagar, Paris, France), and species of isolates confirmed by MALDI-TOF (bioMérieux, Marcy l’Etoile, France). Susceptibility testing followed the EUCAST criteria^16^ using Muller Hinton Agar (Oxoid, Thermo Fisher Scientific, Cambridge, UK) and disc diffusion test (Thermo Fisher Scientific). ESBL production was confirmed by a double-disc synergy test (Mast Group Ltd, Merseyside, UK). Isolates with significant co-resistance to non-beta-lactams were subjected to additional susceptibility testing with Epsilometer tests (ETEST, bioMérieux), disc diffusion test, or broth dilution (Sensititre, Thermo Fisher Scientific).

For the statistical analyses, the urinary isolates with intermediate susceptibility (I) were categorized as susceptible (S). For ESBL-PE UTI patients receiving pivmecillinam, see definition for microbiological (mis)matching.

### Statistical analyses

SPSS Statistics Software (version 28.0.0.0, IBM Corp., USA) and Stata (version 18.0, StataCorp LLC, USA) were used for statistical analyses. *P* values were calculated with the chi-square test.

## Results

Among the 159 592 positive urinary cultures screened, we identified 27 957 samples with Enterobacterales species (1066 ESBL-PE, 26 891 non-ESBL-PE) collected from patients aged 18–65-years. After excluding multiple samples per patient and deceased individuals, we selected all ESBL-PE patients and a corresponding number of non-ESBL-PE patients. In total, 1857 patients were screened for exclusion criteria. For data of excluded/declined patients, see Figure 1. The final study population comprised 130 ESBL-PE UTI and 187 non-ESBL-PE-UTI patients. A total of 184 participants (58.0%) delivered the 3-month follow-up questionnaire.

### Demographics

For demographics, see Table 1. The median age was 51 years, with 89.3% (283/317) females. In total 21.8% (69/317) of the infections were upper UTIs (non-ESBL-PE UTI 16.0%, ESBL-PE UTI 30.0%). Recurrent UTIs were reported by 31.1% (98/317).

**Table 1.** Demographics of participants with UTI caused by either non-ESBL-PE or ESBL-PE over the past 3 months

	All UTI (%) *n* = 317	non-ESBL-PE UTI (%) *n* = 187	ESBL-PE UTI (%) *n* = 130	*P* value
Sex (md = 0)				0.009
Female	283 (89.3)	174 (93.0)	109 (83.8)	
Male	34 (10.7)	13 (7.0)	21 (16.2)	
Age, median (IQR) (md = 0)	51 (35–59)	51 (35–60)	49 (34–57.25)	0.372
Underlying disease or condition (md = 0)	191 (60.3)	119 (63.6)	72 (55.4)	0.140
Cardiovascular or respiratory disease	109 (34.4)	66 (35.3)	43 (33.1)	0.683
Gastrointestinal disease	15 (4.7)	9 (4.8)	6 (4.6)	0.935
Diabetes mellitus	30 (9.5)	21 (11.2)	9 (6.9)	0.190
Renal insufficiency or transplant	5 (1.6)	4 (2.1)	1 (0.8)	0.356
Bladder dysfunction or any urinary catheter	18 (5.7)	13 (7.0)	5 (3.8)	0.225
Immunosuppression	22 (6.9)	12 (6.4)	10 (7.7)	0.661
Recurrent UTIs (md = 2)	98 (31.1)	63 (34.1)	35 (26.9)	0.178
Pathogen (md = 0)				0.477
*E. coli*^a^	298 (94.0)	176 (94.1)	122 (93.8)	
*Klebsiella pneumoniae*^b^	19 (6.0)	11 (5.9)	8 (6.2)	
Upper UTI (md = 0)	69 (21.8)	30 (16.0)	39 (30.0)	0.003

ESBL-PE, ESBL-producing Enterobacterales; UTI, urinary tract infection; md, missing data.

*P* values calculated by chi-square test.

^a^Two co-infections with *Enterococcus faecalis*, one with *K. pneumoniae*, one with *Citrobacter* species, one with *Staphylococcus saprophyticus* and one with *Streptococcus agalactiae*.

^b^One co-infection with *E. coli* and one with *Enterococcus faecalis*.

### Uropathogens and antimicrobial treatment of UTI

As uropathogen, *Escherichia coli* was recorded in 94.0% (298/317) and *Klebsiella pneumoniae* in 6.0% (19/317) of the UTIs. Co-infection rates were 2.0% (6/298) for the *E. coli*, and 10.5% (2/19) for the *K. pneumoniae* UTIs (Table 1).

The most frequently used initial antimicrobials for cystitis were pivmecillinam (33.7%, 63/187 of those with antimicrobial therapy data available), trimethoprim (30.5%, 57/187) and nitrofurantoin (21.9%, 41/187), and for upper UTI ciprofloxacin (34.7%, 17/49), pivmecillinam (14.3%, 7/49), nitrofurantoin (12.2%, 6/49) and cefuroxime (10.2%, 5/49) (Table 2). Data on the initial treatment were missing for 21.8% (54/248) of cystitis and 27.5% (19/69) of upper UTI cases (administration route known); 2.5% (8/317) had not received any antimicrobials.

**Table 2.** Antimicrobial regimens initially used for treatment of patients with non-ESBL-PE or ESBL-PE UTI. Antimicrobials for lower and upper UTI are shown separately

Treatment	All, cystitis/upper UTI (%) *n* = 244^a^	Non-ESBL-PE UTI, cystitis/upper UTI *n* = 147	ESBL-PE UTI, cystitis/upper UTI *n* = 97	*P* value
				0.457/0.015
No antibiotic	7 (3.6)/1 (2.0)	4 (3.2)/0 (0.0)	3 (4.4)/1 (3.4)	
Pivmecillinam^b^	63 (32.5)/7 (14.0)	40 (31.7)/6 (28.6)	23 (33.8)/1 (3.4)	
Trimethoprim	57 (29.4)/3 (6.0)	43 (34.1)/0 (0.0)	14 (20.6)/3 (10.3)	
Co-trimoxazole	4 (2.1)/1 (2.0)	2 (1.6)/1 (4.8)	2 (2.9)/0 (0.0)	
Nitrofurantoin	41 (21.1)/6 (12.0)	25 (19.8)/1 (4.8)	16 (23.5)/5 (17.2)	
Fluoroquinolone	8 (4.1)/18 (36.0)^c^	5 (4.0)/5 (23.8)	3 (4.4)/13 (44.8)	
Cephalexin	9 (4.6)/3 (6.0)	5 (4.0)/3 (14.3)	4 (5.9)/0 (0.0)	
Amoxicillin (±clavulanic acid)	2 (1.0)/0 (0.0)	0 (0.0)/0 (0.0)	2 (2.9)/0 (0.0)	
Cefuroxime	3 (1.5)/5 (10.0)	2 (1.6)/3 (14.3)	1 (1.5)/2 (6.9)	
Ceftriaxone	0 (0.0)/3 (6.0)	0 (0.0)/2 (9.5)	0 (0.0)/1 (3.4)	
Ertapenem	0 (0.0)/3 (6.0)	0 (0.0)/0 (0.0)	0 (0.0)/3 (10.3)	

ESBL-PE, ESBL-producing Enterobacterales; UTI, urinary tract infection.

*P* values calculated by chi-square test.

^a^Detailed data on the initial antimicrobial administered is missing from 73 participants, but the route of administration is known. Peroral regimens were received by 30/3 (cystitis/upper UTI) non-ESBL-PE UTI patients and 22/5 ESBL-PE UTI patients; intravenous regimens were received by 1/6 non-ESBL-PE UTI patients and 1/5 ESBL-PE UTI patients. Intravenous regimens are probably mostly cefuroxime according to Finnish UTI guidelines.^13^

^b^In ESBL-PE UTI group, pivmecillinam dosage data was available for 9/24 patients, of whom seven received the standard dose of 200 mg, and two 400 mg three times a day.

^c^17 ciprofloxacin, 1 levofloxacin

The initial regimen followed the Finnish guidelines for 86.6% (162/187) of those with cystitis and 46.9% (23/49) with an upper UTI.

### Antimicrobial susceptibility

Resistance to non-beta-lactam antimicrobials was more common among ESBL-PE than non-ESBL-PE isolates (Table 3), with the largest differences for fluoroquinolones (*P* < 0.001), trimethoprim (*P* < 0.001) and co-trimoxazole (*P* < 0.001). All non-ESBL-PE isolates and all but one ESBL-PE were susceptible to at least one recommended UTI antimicrobial. Of the 130 ESBL-PE strains, two (1.5%) were carbapenem-resistant.

**Table 3.** The proportion of resistant isolates for various UTI antimicrobials in groups non-ESBL-PE and ESBL-PE UTI

	non-ESBL-PE (%) *n* = 187	ESBL-PE (%) *n* = 130	*P* value
Mecillinam (md = 1)	2 (1.1)	7 (5.4)	0.039
Trimethoprim (md = 0)	38 (20.3)	86 (66.2)	<0.001
Co-trimoxazole (md = 32)	6 (3.9)	85 (65.9)	<0.001
Nitrofurantoin (md = 5)	3 (1.6)	5 (3.9)	0.232
Fluoroquinolones (md = 0)	12 (6.4)	61 (46.9)	<0.001
Cephalexin (md = 20)	1 (0.6)	130 (100.0)	<0.001
Cefuroxime (md = 0)	0 (0.0)	130 (100.0)	<0.001
3. generation cephalosporins (md = 0)	0 (0.0)	130 (100.0)	<0.001
Resistant to all antimicrobials in the primary panel^a^ (md = 20)	0 (0.0)	1 (0.8)	0.820

*P* values calculated by chi-square test.

^a^Cephalexin, cefuroxime, trimethoprim, mecillinam, nitrofurantoin and ciprofloxacin.

### Microbiological matching

The initial antimicrobial matched with the *in vitro* susceptibility for 91.6% (164/179) of the non-ESBL-PE and 46.9% (38/81) of the ESBL-PE UTI patients (*P* < 0.001). For matching rates of individual antimicrobials, see Table 4.

**Table 4.** Microbiological matching and clinical cure rates for various antimicrobials received by non-ESBL-PE and ESBL-PE UTI patients. The data are presented as proportions among all participants receiving the given antimicrobial. In group non-ESBL-PE, 157/187 (84.0%) of UTIs were cystitis and 30/187 (16.0%) upper UTIs; the respective figures in group ESBL-PE were 91/130 (70.0%) and 39/130 (30.0%)

Antimicrobial	Microbiological match		Clinical cure	
	non-ESBL-PE UTI (%)	ESBL-PE UTI (%)	non-ESBL-PE UTI (%)	ESBL-PE UTI (%)
Pivmecillinam	45/56 (97.8)	2/12 (16.7)	34/41 (82.9)	13/24 (54.2)
Trimethoprim/co-trimoxazole	34/45 (75.6)	4/19 (21.1)	31/45 (68.9)	6/18 (33.3)
Nitrofurantoin	25/26 (96.2)	21/21 (100.0)	23/24 (95.8)	15/20 (75.0)
Fluoroquinolones	8/10 (80.0)	8/16 (50.0)	7/10 (70.0)	12/16 (75.0)
Cephalosporins	13/13 (100.0)	0/7 (0.0)	11/14 (78.6)	3/8 (37.5)
Amoxicillin (±clavulanic acid)	N/A	0/2 (0.0)	N/A	2/2 (100.0)
Ertapenem	N/A	3/3 (100.0)	N/A	3/3 (100.0)
Any antibiotic	164/179 (91.6)	38/81 (46.9)	142/172 (82.6)	74/119 (62.2)

.

### Clinical outcome

Clinical cure with the first antimicrobial regimen was more likely in the group non-ESBL-PE UTI (82.6%, 142/172) than in the group ESBL-PE UTI (62.2%, 74/119) (*P* < 0.001). For cure rates of individual antimicrobials, see Table 4.

### Compatibility between microbiological matching and clinical outcome

Most cases with microbiological match were clinically cured: 87.3% (131/150) in group non-ESBL-PE UTI, and 83.3% (30/36) in group ESBL-PE UTI. The respective cure rates among those with microbiological mismatch were 33.3% (5/15) and 41.9% (18/43).

**Figure 2.**
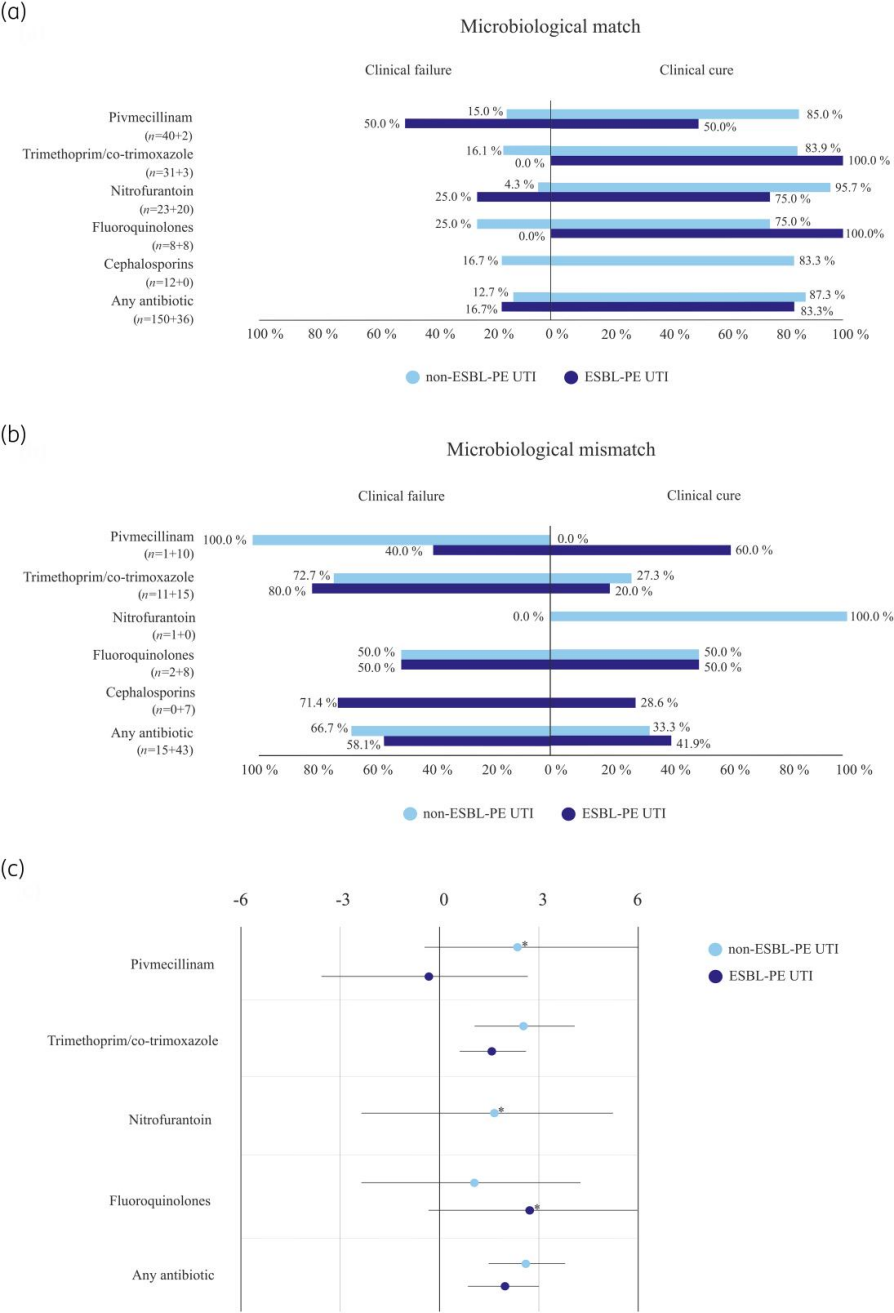
Compatibility of microbiological (a) matching and (b) mismatching with clinical outcome (cure/failure) in non-ESBL-PE or ESBL-PE UTI groups. (c) Forest plot with OR and 95% CI in logarithmic scale comparing clinical failure to microbiological mismatch in both study groups. Note: the validity of each percentage should be evaluated with respect to case numbers (*n*) listed on the left, given separately for non-ESBL-PE and ESBL-PE UTIs. Data are shown for antimicrobials with total *n* > 10. Excluded: asymptomatic (pregnant), *n* = 9; no antimicrobial therapy, *n* = 8; co-infection with discrepant matching results, *n* = 2; spontaneous symptom resolution, *n* = 1. OR and 95% CI could not be calculated for cephalosporins in either group and for nitrofurantoin in group ESBL-PE UTI. *OR and 95% CI calculated by Firth logistic regression analysis. ESBL-PE, ESBL-producing Enterobacterales; UTI, urinary tract infection; N/A, not applicable; OR, odds ratio; CI, compatibility interval.

For compatibility between microbiological matching and clinical outcome with individual antimicrobials, see Figure 2; for compatibility among cystitis patients, see Figure S1 (available as Supplementary data at *JAC-AMR* Online).

### Microbiological mismatch and relapse/reinfection

Among those clinically cured, the initial microbiological mismatch was not associated with reinfection/relapse: during the 3-month follow-up, a reinfection/relapse was recorded for 18.4% (18/98) of those with microbiological match, and 17.6% (3/17) of those with mismatch (OR 1.0, compatibility interval (CI) 95% 0.2–3.7, *P* = 0.943). No statistically significant differences in the reinfection/relapse rates were observed in the subgroups of non-ESBL-PE and ESBL-PE UTI either (data not shown).

## Discussion

Despite UTIs’ great frequency as indication for antimicrobial use,^17^ there are scant data on the impact of discording treatment on infection outcomes. We explored the uropathogen’s resistance rates in a country with low AMR prevalence, focusing on antimicrobial matching and clinical outcomes. Our results reveal that although microbiological matches are associated with clinical cure and mismatches with failure, outcomes cannot be fully anticipated from *in vitro* susceptibility results. Furthermore, we demonstrate that microbiological mismatch is not associated with UTI recurrence.

### Adherence to guidelines

Most (87%) cystitis treatments followed Finnish guidelines.^13^ The substantially lower adherence rate for upper UTI (47%) may be explained by lack of data on fever onset dates, potentially resulting in inclusion of medications initiated before the patients became febrile. Furthermore, due to missing data, many intravenous treatments were recorded as unknown (16%), despite Finnish guidelines having cefuroxime as the standard for patients seeking hospital care.^13^ Therefore, the actual adherence to upper UTI guidelines probably exceeds the recorded rates. Some deviations from guidelines may be clinically justified (e.g. kidney failure, high risk of ESBL-PE infection).

### Resistance rates among uropathogens

For non-ESBL-PE isolates the overall resistance rates were low, paralleling those reported in 2022 for urinary *E. coli* isolates in Finland: 4%, <1%, 6% and 15% for pivmecillinam, nitrofurantoin, fluoroquinolones and trimethoprim, respectively.^18^

For ESBL-PE isolates, our high co-resistance rates for trimethoprim, co-trimoxazole and fluoroquinolones accord with other reports: even in countries with low AMR prevalence, co-resistance rates up to 70% have been reported for these antimicrobials.^3,7^ The high rates may be related to the frequent acquisition of ESBL-PE in travel,^19,20^ and genetic linkage between some resistance genes.^21^ Consistent with our data, low resistance rates have been reported for nitrofurantoin.^21–23^

### Microbiological matching

As expected, the resistance patterns were reflected in the matching results: in accord with the high co-resistance rates among ESBL-PE isolates, we recorded considerably higher microbiological mismatch rates in the ESBL-PE (53%) than the non-ESBL-PE UTI (8%) group (*P* < 0.001).

Among individual antimicrobials, the regimen of pivmecillinam is unique: a higher dose is recommended for ESBL-PE than non-ESBL-PE cystitis (400 versus 200 mg three times a day).^14^ However, when selecting empiric antimicrobials in low-prevalence settings, ESBL-PE is often not considered as a potential pathogen. To address this practice, we classified ESBL-PE UTI treatment as matching only if this higher dose had been used. For pivmecillinam, analysis based on only *in vitro* susceptibility and not dosage (as for other antimicrobials) would have yielded a matching rate of up to 88% as opposed to 17% by the chosen approach.

### Clinical outcomes

Consistent with previous studies,^10,24^ we found higher treatment failure rates for ESBL-PE (38%) than non-ESBL-PE (17%) UTIs (*P* < 0.001), the differences probably attributable to ESBL-PE’s higher co-resistance rates. Our overall clinical failure rate of 17% in non-ESBL-PE UTIs aligns with previous reports with 5–30% for UTIs.^9,10,14,23,25^ For pivmecillinam, the clinical failure rates were higher in ESBL-PE than non-ESBL-PE UTI (46% versus 17%), reflecting underuse of the recommended increased antimicrobial dose in the former group.

### Compatibility between microbiological matching and clinical outcome

Theoretically, microbiological matching (*in vitro* susceptibility to antimicrobial administered) is anticipated to lead to clinical cure, and mismatching to clinical failure. However, in 2002, Rex and Pfaller^26^ described a ‘90–60 rule’ for infections in general, suggesting that 90% of infections treated with matching and 60% of those with mismatching antimicrobials will respond to treatment regardless of pathogen, site of infection or chosen outcome. Consistent with this rule, our data reveal discordance between microbiological matching and clinical outcome, although with slightly different rates: in our non-ESBL-PE UTI group, clinical cure was recorded for 87% of matching and 33% of non-matching cases, and in our ESBL-PE UTI group, for 83% and 42%, respectively. Thus, matching appeared to predict clinical cure better than mismatching predicted failure. An imperfect, yet significant association between microbiological matching and clinical outcome has also been reported by previous UTI studies.^9–11^ Ours is the first to separately examine non-ESBL-PE and ESBL-PE UTI patients, revealing similar compatibility rates for both groups.

The discrepancies between microbiological matching and clinical outcomes may be attributable to several factors. Below, we discuss separately those related to clinical cure despite mismatching antimicrobials and those with clinical failure despite matching antimicrobials.

### Clinical cure despite microbiological mismatch

Clinical cure despite mismatching treatment may be attributed to such factors as susceptibility testing cut-off limits,^16^ high urine concentrations of some antimicrobials^27,28^ and spontaneous recovery of cystitis.^29–32^

Rather than verifying clinical cure rates, EUCAST establishes *in vitro* susceptibility/resistance breakpoints based on factors such as exposure, dose, administration frequency/form, and pharmacokinetics.^16^ Therefore, despite *in vitro* resistance, the urinary drug concentration may reach levels sufficient to kill the bacteria or at least reduce their multiplication.^27,28^ In our data, half of the fluoroquinolone-treated UTIs achieved clinical cure despite microbiological mismatch.

The self-limiting nature of uncomplicated cystitis appears to mainly account for the cure: for 30–70% of patients their cystitis resolves itself within 1 week.^29–32^ Given the low risk of progression to pyelonephritis from uncomplicated cystitis^29–32^ delayed antimicrobial prescription is expected to reduce antimicrobial usage.^33,34^

### Clinical failure despite microbiological match

Clinical failure despite matching antimicrobial treatment may stem from such factors as inappropriate cut-off limits in susceptibility testing^16^ (see above), heteroresistance,^35^ non-compliance, antimicrobial’s insufficient tissue penetration^4,5^ or host factors.^9–11,26,36–38^

The lack of efficacy perceived for pivmecillinam and nitrofurantoin may be partly attributed to their limited tissue penetration,^4,5^ particularly among the 12.5% of patients with upper UTI. However, this does not fully explain it, as compatibility rates among cystitis patients mirrored those of all UTI patients. Separate analyses for upper UTI were not conducted due to the small sample size.

Host factors probably account for a great deal of clinical failures despite matching antimicrobial treatment. Factors such as advanced age, male sex, comorbidities and organ dysfunction have been associated with clinical UTI treatment failure.^9–11,36–38^ However, because of scant research, the precise determinants of UTI outcomes beyond microbiological factors remain unclear.

### (Mis)matching antimicrobials and relapse/reinfection

Microbiological (mis)match did not affect UTI reinfection/relapse risk, consistent with studies reporting similar recurrence rates for those administered antimicrobials of varying efficacy.^8,39^ Treating a single UTI episode successfully appears thus not to prevent recurrences.

The lack of correlation between microbiological matching and recurrences appears logical, since only part (18–85%) of the recurring UTIs are caused by the same uropathogen as the previous episode.^40^ Additionally, the treatment is not expected to eradicate the uropathogen from patients’ intestines,^4,5^ and some uropathogenic *E. coli* strains may, by colonizing the urothelium, evade antimicrobials even in the urinary bladder.^1,40^ Interestingly, recently administered antimicrobial therapies have been associated with an increased risk of UTI,^12,41^ suggesting that they may even increase recurrence risk.

### Limitations

As the UTI guidelines advise no urinary cultures for uncomplicated cystitis,^4,5^ our recruitment approach, relying on positive urine cultures, may have overrepresented complicated, recurrent or upper UTIs, or those only sampled after failure with the primary treatment, seen as higher resistance rates.

To further explore the association between microbiological (mis)matching and clinical outcome, subgroup analyses would have been useful.^42^ However, due to limited sample size, subgroup analyses were only feasible for cystitis patients.

Dates of antimicrobial therapy initiation were available for 179/317 participants. Among these antimicrobials, 22.3% were commenced ≥2 days after sampling. Occasionally, antimicrobials may thus have been chosen on the basis of culture results, potentially reflected as lower microbiological mismatch rates and poorer guideline adherence than if all infections had been treated empirically.

Our data lacked explanations for changes in antimicrobial treatment (adverse effects, ineffectiveness, other), which may have been reflected as slightly higher clinical failure rates.

Finally, recording data afterward may have brought on recall bias. If available, we retrieved data on antimicrobial treatments from patient records.

### Conclusions

Our study contributes valuable insights to the scant literature on antimicrobial matching and UTI outcomes, particularly in ESBL-PE UTI. Our data show that the association between microbiological (mis)match and clinical outcome is not flawless; matching appears to predict a clinical cure better than mismatching predicts a failure. Given the clinical importance of identifying patients at risk of clinical failure despite matching antimicrobial therapy, further research is warranted to elucidate the risk factors for UTI treatment failure.

## Supplementary Material

dlae188_Supplementary_Data

## Data Availability

All relevant data are in the paper and its Supplementary material file. The individuals’ data cannot be shared publicly due to research regulations. Further information directly from the authors.

## Supplementary data

Figure S1 is available as Supplementary data at *JAC-AMR* Online.
