# Supplementary material for: Urinary tract infections: a retrospective cohort study of (mis)matching antimicrobial therapy and clinical outcome among Finnish adults
Source: JAC Antimicrob Resist. 2024 Nov 26;6(6):dlae188. doi: 10.1093/jacamr/dlae188 (PMC11589465; doi:10.1093/jacamr/dlae188)
Supplement: dlae188_Supplementary_Data [file dlae188_supplementary_data.docx]

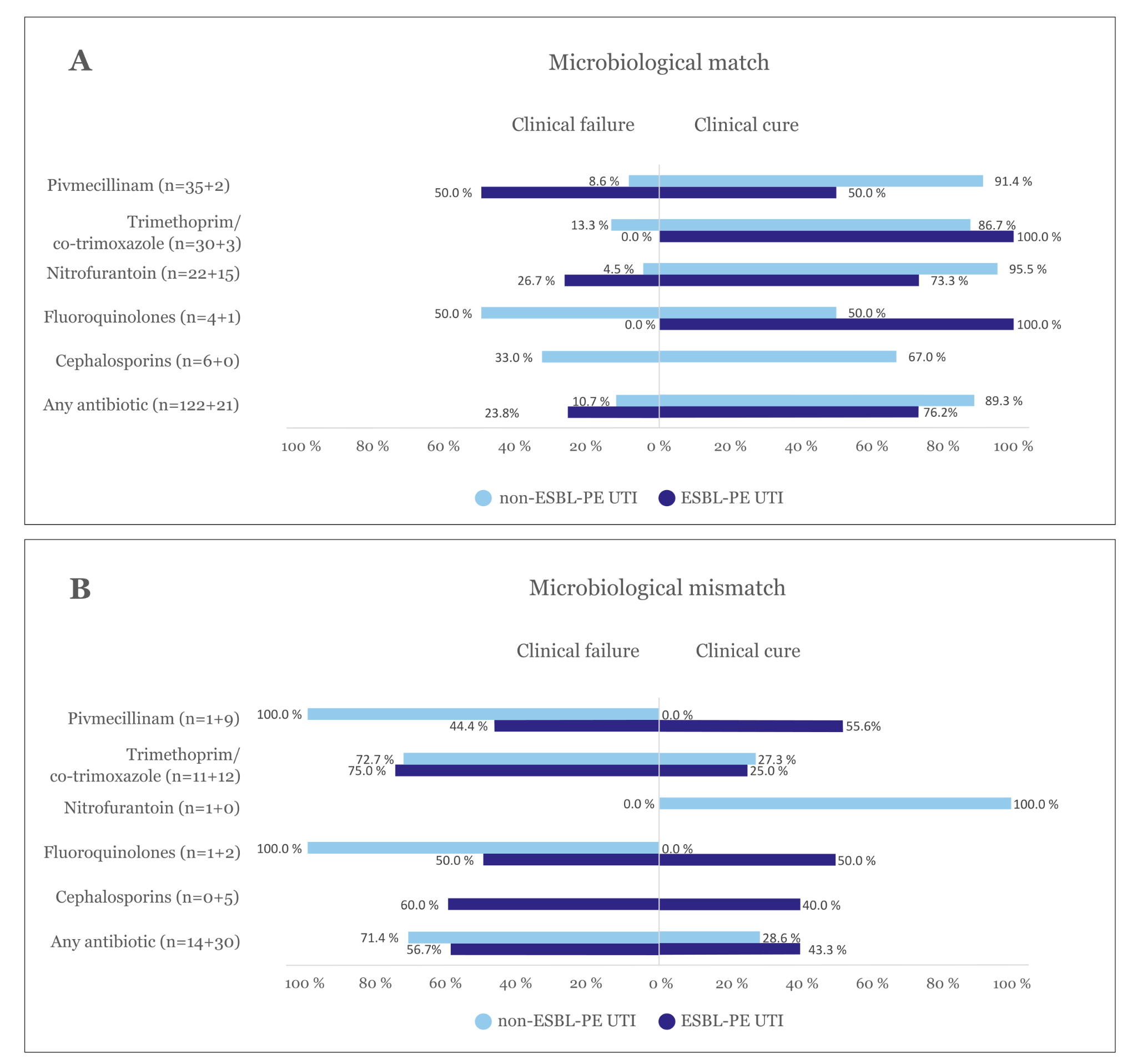
**Supplementary Figure S1.** Compatibility of microbiological A) matching and B) mismatching with clinical outcome (clinical failure/cure) among patients with non-ESBL-PE or ESBL-PE cystitis.

Note: the validity of each percentage should be evaluated with respect to case numbers (n) listed on the left, given separately for non-ESBL-PE and ESBL-PE UTIs.

Excluded: asymptomatic (pregnant), n=9; no antimicrobial therapy, n=7; co-infection with discrepant matching results, n=2; spontaneous symptom resolution, n=1.

Abbreviations: ESBL-PE, ESBL-producing Enterobacterales; UTI, urinary tract infection
